# The precancer risk of betel quid chewing, tobacco use and alcohol consumption in oral leukoplakia and oral submucous fibrosis in southern Taiwan

**DOI:** 10.1038/sj.bjc.6600727

**Published:** 2003-02-10

**Authors:** C-H Lee, Y-C Ko, H-L Huang, Y-Y Chao, C-C Tsai, T-Y Shieh, L-M Lin

**Affiliations:** 1 Graduate Institute of Public Health, College of Health Science, Kaohsiung Medical University, Taiwan, ROC; 2 Graduate Institute of Medicine, College of Medicine, Kaohsiung Medical University, Taiwan, ROC; 3 Department of Nursing, Shu-Zen College of Medicine and Management, Taiwan, ROC; 4 Graduate Institute of Dentistry, College of Dentistry, Kaohsiung Medical University, Taiwan, ROC; 5 Graduate Institute of Oral Health Sciences, College of Dentistry, Kaohsiung Medical University, Taiwan, ROC

**Keywords:** oral leukoplakia, oral submucous fibrosis, risk factors, areca, smoking, alcohol drinking

## Abstract

In areas where the practise of betel quid chewing is widespread and the chewers also often smoke and drink alcohol, the relation between oral precancerous lesion and condition to the three habits is probably complex. To explore such association and their attributable effect on oral leukoplakia (OL) and oral submucous fibrosis (OSF), a gender–age-matched case–control study was conducted at Kaohsiung, southern Taiwan. This study included 219 patients with newly diagnosed and histologically confirmed OL or OSF, and 876 randomly selected community controls. All information was collected by a structured questionnaire through in-person interviews. A preponderance of younger patients had OSF, while a predominance of older patients had OL. Betel quid chewing was strongly associated with both these oral diseases, the attributable fraction of OL being 73.2% and of OSF 85.4%. While the heterogeneity in risk for areca nut chewing across the two diseases was not apparent, betel quid chewing patients with OSF experienced a higher risk at each exposure level of chewing duration, quantity and cumulative measure than those who had OL. Alcohol intake did not appear to be a risk factor. However, cigarette smoking had a significant contribution to the risk of OL, and modified the effect of chewing based on an additive interaction model. For the two oral premalignant diseases combined, 86.5% was attributable to chewing and smoking. Our results suggested that, although betel quid chewing was a major cause for both OL and OSF, its effect might be difference between the two diseases. Cigarette smoking has a modifying effect in the development of oral leukoplakia.

Studies from Pakistan, India and Mainland China consistently showed that chewing of areca nut was the major aetiologic factor for oral leukoplakia (OL) and oral submucous fibrosis (OSF) (Mehta *et al*, 1981; Maher *et al*, 1994; Tang *et al*, 1997). However, in a review of case series, the proportion of areca nut chewers among individuals with OL and OSF varied from 43–68% and 34–100%, respectively (Bhonsle *et al*, 1987; Ikeda *et al*, 1995; Hashibe *et al*, 2000). Factors other than areca nut chewing might play a role in the development of oral precancerous diseases in populations.

Tobacco smoking and alcohol abuse are involved in the pathogenesis of oral cavity cancer, and the two agents appear to act synergistically (Blot *et al*, 1988; Merletti *et al*, 1989; Ko *et al*, 1995). Although OL and OSF are both high-risk preneoplastic states, the independent and interactive associations between cigarette smoking, alcohol consumption and areca nut chewing have not been well established in these oral diseases.

In Taiwan, about 2 million people practise the habit of chewing betel quid. As a high proportion of betel quid chewers are also smokers (86%) or drinkers (74%) in southern Taiwan (Ko *et al*, 1992), we present a case–control study investigating the independent and synergistic effects of betel quid chewing, tobacco use and alcohol consumption in the development of OL and OSF, examining the heterogeneity of risk across both the diseases.

## MATERIALS AND METHODS

### Subject selection and data collection

The study population is composed of residents of the greater Kaohsiung area of Taiwan, which includes a city and some suburban and rural communities. The oral precancerous cases in the study were recruited from Kaohsiung Medical University Hospital, which is a highly regarded teaching hospital in tropical southern Taiwan, and is accessible to patients from all socioeconomic groups. Subjects who visited the hospital's dentistry department during 1994 and 1995 and were suspected of having OL or OSF on clinical criteria were considered as potential cases. Only patients who were newly diagnosed and were histologically confirmed with the two types of oral diseases by pathologists were included. However, patients who showed symptoms of both OL and OSF were excluded. Among the 219 oral precancerous patients, 125 cases (57.1%) suffered from OL and 94 cases (42.9%) suffered from OSF.

The controls were selected randomly through a three-step sampling scheme from a population aged 15 years and over in the greater Kaohsiung area. First, we stratified 38 study areas into two strata by levels of urbanization. Then, 19 study areas were chosen randomly from the two strata before selecting 1864 households from these areas. The number of study areas and households sampled from each stratum were probably proportional to their population size. Finally, one individual was randomly selected from each household. Information regarding the age and gender of the selected subjects was verified through telephone interviews. Once a case was identified, four controls matched by age (within 3 years) and gender were selected according to their sequence on the list. If a selected subject refused to participate in the study, the next eligible person on the list would be selected until four controls were recruited. Of these, 184 subjects refused to, or could not, participate in the study. The reasons were: too busy for the interview; out of town; and moving out and could not be located. A total of 876 matched controls participated in the study.

The research workers were trained in the management of oral precancerous cases and controls. Each subject was interviewed face to face about demographic information, occupations, betel quid chewing, smoking history and alcohol drinking habits with a structured questionnaire. Subjects who had chewed one betel quid or more or had smoked one cigarette or more per day for at least 1 year were defined as ever chewers or ever smokers. Subjects who had drunk a bottle of alcoholic beverages (including beer, liquor and wine) or more per month for at least 1 year were defined as ever drinkers. Among them, current users were those who had practised these habits within the past 1 year, and ex-users were those who had stopped the habits for at least 1 year before diagnoses or interviews. For all of the ever chewers and ever smokers, a detailed history of their chewing and smoking habits was recorded, including daily consumption, age of commencement and duration of practice. For ever drinkers, information of the frequency of alcohol intake was collected. To assess the cumulative risks of betel quid chewing and cigarette smoking, the number of ‘pack-years’, calculated by multiplying the amount (in packs; 20 cigarettes and 10 betel quids per pack) consumed daily by the years of using, was employed as the indicators of chewing and smoking. In addition, the types of regularly chewed betel materials were recorded as follows: areca nut with a piece of inflorescence of Piper Betel Linn, areca nut with a piece of betel leaf, and both mixed.

### Statistical analyses

Odds ratios (ORs) and 95% confidence intervals (CI) were estimated for precancer risk of various factors by using conditional logistic regression analyses. Statistical significance of trend was calculated by categorizing exposure variables and treating scored variables as continuous. Separate analyses were conducted for OL and OSF. To control the potential confounding effects, ORs were adjusted for educational level (<7, 7–9, >9 years) and occupation (white collar, farmers, blue collar). The homogeneity of ORs across the two oral preneoplastic states was examined by the Mantel–Haenszel *χ*^2^ test, and the strength of heterogeneity in ORs between OSF and OL was expressed as OR ratios (Lee *et al*, 2001). Interactive effects of any suspected risk factors were evaluated by assuming an additive interaction relation. The synergism index (SI) proposed by Rothman and its 95% CI were computed to assess the empirical deviation from the additive interaction relation (Hosmer and Lemeshow, 1992). Also, the proportion of oral premalignant cases attributable to one or all risk factors considered (population attributable risk percent; PAR%) was calculated according to Bruzzi *et al*'s (1985) method.

## RESULTS

The demographic distributions of OL and OSF patients, and their controls are presented by five characteristics in Table 1

**Table 1:** Distributions and odds ratios of OL and OSF associated with demographic factors, Taiwan

	**Oral leukoplakia**			**Oral submucous fibrosis**		
**Factor/category**	**No. of cases**	**No. of controls**	**OR (95% CI)**	**No. of cases**	**No. of controls**	**OR (95% CI)**
*Age (years)*						
<31	5	20		22	88	
31–40	37	148		38	152	
41–50	31	124		16	64	
>50	52	208		18	72	
						
Gender						
Male	118	472		93	372	
Female	7	28		1	4	
						
Ethnicity						
Fukienese	97	378	1.0	71	287	1.0
Mainlander	14	54	1.0 (0.5–2.1)	10	35	1.1 (0.5–2.5)
Hakka	11	65	0.7 (0.3–1.3)	11	50	0.9 (0.4–1.8)
Aborigines	3	3	3.9 (0.8–19.6)	2	4	2.2 (0.3–13.4)
						
*Years of education*						
<7	74	259	1.0	37	110	1.0
7–9	21	84	0.8 (0.4–1.4)	19	78	0.6 (0.3–1.2)
>9	30	157	0.6 (0.3–0.9)	38	188	0.4 (0.2–0.8)
						
Occupation						
Blue collar	77	269	1.0	56	183	1.0
Farmer	25	90	1.0 (0.6–1.7)	15	49	1.1 (0.5–2.2)
White collar	23	141	0.6 (0.3–0.9)	23	144	0.5 (0.3–0.9)

. Cases and controls in each disease group were closely matched in age and gender. However, the average diagnostic age of subjects suffering from OL (47.9±11.8 years) was significantly higher than that of subjects suffering from OSF (39.1±11.7 years). Education and occupation were both associated with these two oral diseases. Patients who were white-collar workers or had higher education levels (>9 years) had a lower risk of oral preneoplastic states (OR=0.4–0.6).

Table 2

**Table 2:** Odds ratios of OL and OSF associated with cigarette smoking and alcohol drinking, Taiwan

	**Oral leukoplakia**			**Oral submucous fibrosis**		
**Factor/category**	**No. of cases**	**No. of controls**	**OR^a^ (95% CI)**	**No. of cases**	**No. of controls**	**OR^a^ (95% CI)**
*Cigarette smoking*						
Never^b^	19	258	1.0	10	188	1.0
Ex	6	16	5.6 (1.9–16.6)	5	14	6.5 (1.9–22.3)
Current	100	226	6.1 (3.4–10.6)	79	174	7.0 (3.5–14.3)
Dose–response			2.4 (1.8–3.1)			2.5 (1.8–3.5)
						
*Cumulative pack-years*						
1–10	32	84	5.3 (2.7–10.4)	36	95	5.7 (2.6–12.3)
11–20	26	66	5.4 (2.7–10.9)	20	41	8.6 (3.6–20.7)
>20	48	92	7.0 (3.7–13.2)	28	52	8.6 (3.7–20.2)
Dose–response			1.8 (1.5–2.1)			2.0 (1.5–2.5)
						
*Alcohol drinking*						
Never^b^	72	349	1.0	55	266	1.0
Ex	9	40	1.1 (0.5–2.4)	7	27	1.4 (0.6–3.4)
Current	44	111	1.8 (1.1–2.8)	32	83	1.8 (1.1–3.1)
Dose–response			1.3 (1.1–1.7)			1.4 (1.0–1.8)
						
*Drinking frequency*						
Monthly	9	40	1.1 (0.5–2.4)	7	27	1.4 (0.6–3.4)
Weekly	24	60	1.9 (1.1–3.3)	17	42	1.9 (1.0–3.7)
Daily	20	51	1.7 (0.9–3.0)	15	41	1.7 (0.9–3.5)
Dose–response			1.2 (1.0–1.5)			1.2 (1.0–1.5)

a Odds ratios were adjusted for education and occupation.

b Never smokers and never drinkers were reference categories, respectively.

shows the risks of contracting OL and OSF for cigarette smoking and alcohol drinking. Ex-smokers and current smokers were found to experience, respectively, a 5.6–6.5-fold and 6.1–7.0-fold elevated risk of OL and OSF. However, for subjects with a drinking habit, only current drinkers experienced a higher risk of developing the two oral diseases (OR=1.8 for both diseases). As smoking levels and drinking frequency increased, the risk for developing OL and OSF also increased. A dose–response relation between exposure levels and oral precancer risks was also evidenced (*P*<0.05).

The characteristics of betel quid chewing history were examined in the case–control pairs for OL and OSF (Table 3

**Table 3:** Odds ratios of OL and OSF associated with betel quid chewing, Taiwan

	**Oral leukoplakia**		**Oral submucous fibrosis**		
**Betel quid chewing/category**	**Cases/controls**	**OR^a^ (95% CI)**	**Cases/controls**	**OR^a^ (95% CI)**	**OR^b^ ratio**
Never^c^	28/390	1.0	11/302	1.0	
Ex	6/22	7.1 (2.3–21.5)	5/12	12.1 (2.8–51.9)	
Current	91/88	22.3 (11.3–43.8)	78/62	40.7 (16.0–103.7)	
Dose–response		4.6 (3.3–6.4)		6.2 (3.9–9.7)	1.3
					
*Age first chewed (years)*					
⩾26	51/56	20.6 (9.9–42.7)	37/34	32.3 (12.1–86.6)	
<26	46/54	19.5 (9.3–41.0)	46/40	39.4 (14.8–105.3)	
Dose–response		4.3 (3.1–6.0)		5.8 (3.8–8.8)	1.3
					
*Duration of chewing (years)*					
1–10	33/48	15.9 (7.1–35.6)		30.9 (11.3–84.7)	
11–20	27/33	20.7 (8.9–48.2)	36/36	41.9 (14.1–124.9)	
⩾21	37/29	24.0 (10.8–53.4)	28/25	39.3 (11.7–131.7)	
Dose–response		3.0 (2.3–3.9)	19/13	4.2 (3.0–6.1)	1.4
					
*Quantity of chewing (pieces/day)*					
1–10	53/73	16.6 (8.2–33.8)	41/42	31.4 (11.9–82.5)	
11–20	24/25	21.0 (8.9–49.7)	24/21	37.4 (12.6–110.4)	
⩾21	20/12	38.5 (14.1–105.1)	18/11	53.5 (16.4–174.8)	
Dose–response		3.8 (2.8–5.1)		4.1 (2.9–5.8)	1.1
					
*Cumulative pack-years*					
1–10	33/64	12.0 (5.6–25.7)	35/41	26.5 (10.0–70.3)	
11–20	17/15	23.7 (9.1–61.7)	21/16	47.0 (15.8–139.8)	
⩾21	47/31	31.4 (14.2–69.2)	27/17	51.4 (16.5–159.7)	
Dose–response		3.1 (2.4–3.9)		4.1 (2.9–5.8)	1.3
					
*Types of material*					
With betel inflorescence	60/56	24.5 (11.8–50.7)	47/38	38.7 (14.7–101.9)	1.6
With betel leaf	11/24	11.5 (4.2–32.0)	8/15	18.7 (5.3–66.1)	1.6
Mixed	26/30	17.4 (7.6–39.8)	28/21	37.4 (13.1–107.2)	2.1

a Odds ratios were adjusted for education and occupation.

b The heterogeneity in ORs between OSF and OL was expressed as OR ratio.

c Never chewers were a reference category.

). The risks for the two oral premalignant diseases among current chewers were 22.3–40.7-fold higher than that among never chewers, and only 7.1–12.1-fold increases were observed among ex-chewers. The heterogeneity in risk (expressed as an OR ratio) between the two oral diseases was nonsignificant (*P*>0.05). At each exposure level of chewing duration, quantity and cumulative measure, the betel quid chewing patients with OSF had a higher precancer risk than those with OL. Furthermore, the risk did not relate to the types of betel materials. Chewing betel quid with a piece of Piper Betel Linn inflorescence showed the highest precancer risk in both the oral diseases.

The synergistic effects of betel quid chewing, cigarette smoking and alcohol drinking on OL and OSF were evaluated by stratifying the uses of tobacco and alcohol across the habit of betel quid chewing (Table 4

**Table 4:** Synergistic effects of OL and OSF between betel quid chewing, cigarette smoking and alcohol drinking, Taiwan

	**Oral leukoplakia**			**Oral submucous fibrosis**			
**Factors/category**	**Cases/controls**	**OR (95% CI)**	**SI^a^ (95% CI)**	**Cases/controls**	**OR (95% CI)**	**SI^a^ (95% CI)**	**OR^b^ ratio**
*Betel chewing/cigarette smoking^c^,^d^*							
No/no	12/235	1.0		4/178	1.0		
No/yes	16/155	2.4 (1.0–5.5)		7/124	2.3 (0.6–9.1)		1.0
Yes/no	7/23	10.0 (3.1–32.7)		6/10	39.3 (7.5–206.9)		3.9
Yes/yes	90/87	40.2 (16.3–99.2)	3.8 (1.4–10.5)	77/64	57.9 (16.0–209.6)	1.4 (0.4–4.7)	1.4
							
*Betel chewing/alcohol drinking^c^,^e^*							
No/no	22/292	1.0		9/223	1.0		
No/yes	6/98	1.0 (0.4–2.6)		2/79	0.7 (0.1–3.4)		0.7
Yes/no	50/57	15.6 (7.1–34.3)		46/43	26.5 (9.5–74.1)		1.7
Yes/Yes	47/53	16.8 (7.2–39.5)	1.1 (0.6–2.1)	37/31	31.7 (10.1–99.3)	1.2 (0.6–2.5)	1.9

a Synergism index estimated by an additive interaction model.

b The heterogeneity in ORs between OSF and OL was expressed as OR ratio.

c ‘Yes’ referred to the ‘ever users’ for betel quid chewing, cigarette smoking and alcohol drinking.

d Odds ratios were adjusted for education, occupation and alcohol drinking.

e Odds ratios were adjusted for education, occupation and cigarette smoking.

). For nonsmokers and nondrinkers who practised the habit of betel quid chewing, the risks of oral OSF increased 39.3- and 26.5-fold, respectively, compared with those who did not have the habit. The risks were largely increased for areca nut chewers who also have the habit of smoking or drinking (OR=57.9 and 31.7). Similar risk patterns were observed among the patients of OL. However, for smokers who did not chew betel quid, the significant risk of OL was detected. Moreover, cigarette smoking was found to modify the effect of betel quid chewing based on the model of additive interaction (SI=3.8; *P*<0.05). Although most of the OR ratios between OSF and OL for areca nut chewing alone or combined with smoking or drinking were larger than one, no statistical heterogeneity was identified.

Multivariate logistic regression analyses were separately conducted for OL, OSF and the two diseases combined (Table 5

**Table 5:** Adjusted odds ratios and population attributable risk proportions (PAR%) of OL and OSF associated with independent factors, Taiwan

	**Oral leukoplakia**		**Oral submucous fibrosis**	
**Factor/category**	**OR^a^ (95% CI)**	**PAR%**	**OR^a^ (95% CI)**	**PAR%**
*Cumulative betel quid chewing in pack-years*				
No	1.0	73.2	1.0	85.4
1–10	10.2 (4.6–23.1)		22.4 (8.0–62.3)	
11–20	24.5 (8.8–68.1)		40.4 (12.9–126.6)	
⩾21	28.8 (11.9–69.5)		44.0 (12.8–151.8)	
				
*Cumulative cigarette smoking in pack-years*				
No	1.0	56.4	1.0	
1–10	3.3 (1.5–7.2)		1.8 (0.7–5.1)	—
11–20	3.0 (1.3–7.2)		2.0 (0.6–6.4)	
⩾21	2.8 (1.3–6.0)		1.6 (0.5–5.0)	
				
*Alcohol drinking*				
No	1.0	—	1.0	—
Monthly	1.0 (0.4–2.7)		0.8 (0.3–2.5)	
Weekly	1.1 (0.5–2.2)		1.3 (0.5–3.1)	
Daily	0.8 (0.4–1.7)		0.9 (0.3–2.5)	
				
*Years of education*				
<7	1.0		1.0	
7–9	1.6 (0.8–3.2)	—	0.7 (0.3–1.8)	—
>9	1.0 (0.5–2.1)		0.6 (0.2–1.7)	
				
*Occupation*				
Blue collar	1.0	—	1.0	—
Farmer	0.6 (0.3–1.2)		0.5 (0.2–1.4)	
White collar	1.1 (0.6–2.2)		1.0 (0.4–2.1)	
				
				
Summary population attributable risk proportion (%)		84.4		85.4

a Odds ratios were derived from the multivariate logistic regression model adjusted for the table's covariates.

, Figure 1

**Figure 1:**
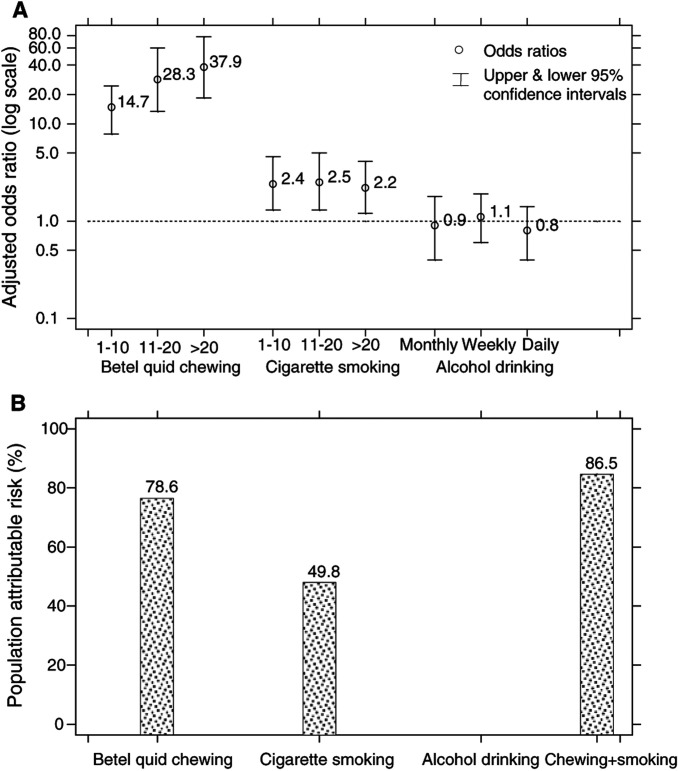
**(A)** Adjusted ORs and CIs, and **(B)** population attributable risk proportions of OL and OSF combined associated with betel quid chewing, cigarette smoking and alcohol drinking, Taiwan.

). Betel quid chewing was found to be the strongest risk factor for both OL (OR>10) and OSF (OR>22). While the effect of alcohol drinking on the two oral premalignant diseases was not substantial, the significantly elevated risk of cigarette smoking was detected among OL patients. Among these risk factors, betel quid chewing accounted for 73.2 and 85.4% of attributable risks of contracting OL and OSF, respectively. Combined with cigarette smoking, the population attributable risk proportion of OL increased to 84.4%. In the same way, 86.5% of the aaetiologic fraction for patients having either OL or OSF was detected (Figure 1).

## DISCUSSION

This study found that betel quid chewing was the principal cause of OL and OSF. Subjects who ever chewed areca nut experienced a more than 11-fold risk of these precancerous conditions. The risks increased with the duration and frequency of the habit, as previously shown in Pakistan, India, Taiwan and Mainland China (Mehta *et al*, 1981; Maher *et al*, 1994; Tang *et al*, 1997; Shiu *et al*, 2000).

The chewing of betel quid is practised in several different ways in various countries, while the major components are comparatively consistent. In India and Southeast Asia, tobacco was usually used as an ingredient for areca nut products (called ‘pan’), but not in Taiwan. A higher relative risk of oral cancer for betel quid chewing with tobacco was notably higher than that for betel quid chewing without tobacco, and the evidence for OL was also in the same direction (Gupta *et al*, 1982). Our study showed that non-smokers and nondrinkers who chewed betel quid had, respectively, a 10.0–15.6- and 26.5–39.3-fold significant risk of OL and OSF (Table 4), and both risks were lower than that reported for tobacco-contained areca nut products (OR=17.4 and 44.1 for OL and OSF, respectively) (IARC, 1985; Hashibe *et al*, 2000). The difference in risks between areca nut with and without tobacco implies that tobacco could have an additional effect on OL and OSF.

Significantly elevated risks of OL and OSF were registered at the lowest levels of betel chewing quantity (1–10 pieces day^−1^) and duration (1–10 years). The data indicated that even a relatively short exposure is sufficient to induce leukoplakia or mucous fibrosis, as previously suggested (Seedat and Van Wyk, 1988; Maher *et al*, 1994). Arecoline, the most abundant alkaloid in areca nut, has been observed experimentally to stimulate collagen synthesis by fibroblasts *in vitro* (Canniff and Harvey, 1981). Studies of human buccal fibroblasts found that arecoline was not only cytotoxic but stimulated double-stranded polynucleic acid synthesis; both might act synergistically on the pathogenesis of OSF as well as oral cancer (Chang *et al*, 1998).

OL and OSF are clinically distinct precancerous lesions that precede the development of oral cancer. Our study showed that the risk of OSF at each exposure level of betel quid chewing was stronger than those of OL, although the difference was not large enough to reject the null. Similar results were found in large-scale case–control studies conducted in India (Hashibe *et al*, 2000,^2002^). We also found that mainly younger patients had OSF compared with mainly older patients with OL. The fact that OSF patients started betel quid chewing at a younger age than OL patients and chewed more quids per day may partly explain the age differences between the two diseases.

Our multivariate analyses indicated that cigarette smoking was an independent risk factor for OL, but not for OSF. While the associations between tobacco smoking and the two types of oral premalignant diseases have not been definitely established, comparable findings were observed in India and Europe (Banoczy *et al*, 2001; Hashibe *et al*, 2000,^2002^). We found a significant precancer risk of cigarette smoking among OL patients who did not chew betel. In contrast, the effects of betel quid chewing alone on OSF among nonsmokers and nondrinkers were much higher than those on OL (OR ratios ⩾1.7), reflecting the substantial role of smoking in OL, although the effect of betel quid chewing is much stronger on OSF, as discussed earlier. In addition, it has been noticed that the risk of OL and OSF is greatly increased in the presence of both betel quid chewing and smoking (Table 4). Cigarette smoking was found to modify the effect of betel quid chewing in OL based on an additive interaction model. However, the joint risk of OSF for the two factors was still higher than the combined risk of OL, assessed by multiple logistic regression models.

Although ethanol has been recognized as a solvent that may damage the oral cells and increase the mucosal penetration of certain oral carcinogens (Hashibe *et al*, 2000), the role of alcohol drinking in the development of OL is still unclear. In a cross-sectional study, an independent effect of alcohol use on OL was not identified (Gupta, 1984) nor in Uzbekistan (Evstifeeva and Zaridze, 1992). In contrast, studies in Kenya (Macigo *et al*, 1996) and India (Hashibe *et al*, 2000) suggested that drinking was a moderate risk factor, and a clear dose–response relation between alcohol consumption and OL was evidenced. In our study, alcohol intake was not associated with OL. Among OL patients with precancerous lesion, 88.7 and 98.1% of alcohol users were also betel quid and tobacco consumers, respectively. The nonsignificant risks in the multiple regression models indicated that the effect of drinking was explained by betel quid chewing and cigarette smoking. Alcohol use was not an important risk factor for OL in our southern Taiwan population. On the other hand, our study indicated that alcohol consumption was not related to OSF. This result was consistent with the findings from previous studies (Maher *et al*, 1994; Yang *et al*, 2001).

Oral cancer has been one of the 10 leading causes of cancer deaths in Taiwan since 1982. The mortality of oral cancer increased about 2.6-fold from 1971 to 1997 (Department of Health (ROC), 1998), making its prevention an important public health issue in Taiwan. Since it is often preceded by OL and OSF, and the cessation of areca nut chewing has been associated with a regression in the incidence of OL (Gupta *et al*, 1995), study of their risk factors and their population attributable risk proportion may allow better directed prevention efforts. Our study showed that 73.2 and 56.4% of the aetiologic fraction of OL were, respectively, attributable to betel quid chewing and cigarette smoking. In contrast, the habit of chewing betel quid accounted for 85.4% of attributable risk of OSF. Additionally, it is reasonable to expect that the avoidance of chewing and smoking may possibly prevent 86.5% of the two oral premalignant diseases, and thereby be of considerable health benefit to Taiwan.

One concern in this study is that the oral cavity status of controls was not examined. Since the incidence of the two oral diseases was relatively low, the bias resulting from the inclusion of possible cases in the control group should be limited, and should, if anything, tend to underestimate the risks.

In summary, the chewing of betel quid significantly contributed to the risks of having OL and OSF, and an overwhelming majority were attributable to the practise of areca nut chewing. Cigarette smoking also had a substantial role in the occurrence of OL and potentiated the effect of betel quid chewing.
